# Implementing a smoking cessation training for lower socioeconomic groups into local policy in Amsterdam, the Netherlands: Identifying preconditions and barriers among multiple stakeholder groups

**DOI:** 10.1093/eurpub/ckac129.756

**Published:** 2022-10-25

**Authors:** J Harting, MAG Kuipers

**Affiliations:** Department of Public and Occupational Health, Amsterdam UMC, University of Amsterdam, Amsterdam, Netherlands

## Abstract

**Background:**

Socioeconomic inequalities in smoking prevalence are high, partly because smoking cessation programs are insufficiently accessible and suitable for smokers with a lower socioeconomic position (SEP). To make it easier for this target group to access suitable smoking cessation programs, it is necessary to structurally implement such programs into local policies. The study aims to identify preconditions and barriers for the implementation of smoking cessation programs for people with low SEP from the perspective of key stakeholders.

**Methods:**

The Feel Free! Smoking cessation rolling group training has been previously developed for people from lower socio-economic groups and was implemented in Amsterdam Noord. Semi-structured interviews were held with 25 stakeholders consisting of participants, trainers, professionals in the neighbourhood and stakeholders of the municipality. The interviews were audio-recorded, transcribed verbatim and analysed using a thematic approach. The Implementation Framework of Fleuren will be used to structure the presentation.

**Results:**

The main preconditions found are effective recruitment of participants by local professionals, having a central coordinator for implementation within the neighbourhood network, and offering a smoking cessation program with a clear added value for participants. The main barriers found are challenges in setting up a sustainable financial structure, allocation of organizational tasks, and high participant absences and dropout. More results will be presented in detail.

**Conclusions:**

This study shows that action is required from various stakeholders to facilitate the implementation process. These findings can inform policy makers and implementers to choose strategies to implement suitable smoking cessation programs into local policy.

